# Difficulties in demonstrating long term immunity in FeLV vaccinated cats due to increasing age-related resistance to infection

**DOI:** 10.1186/1746-6148-8-125

**Published:** 2012-07-28

**Authors:** Stephen Wilson, Juliet Greenslade, Gillian Saunders, Catherine Holcroft, Lynn Bruce, Andy Scobey, Tedd Childers, Gordon Sture, James Thompson

**Affiliations:** 1 Veterinary Medicine Research and Development, Pfizer Animal Health, Pfizer European Service Centre, Zaventem, Belgium; 2 Veterinary Medicine Research and Development, Pfizer Animal Health, Ramsgate Road, Sandwich, UK; 3 Pfizer Animal Health, Portage Road, Kalamazoo, USA

**Keywords:** Feline, Leukaemia, Vaccine, Protection

## Abstract

**Background:**

Feline leukaemia virus (FeLV) is a pathogen causing fatal illness in cats worldwide, and as such there is a high demand for products to protect against disease. The duration of immunity provided by an inactivated FeLV vaccine, Versifel FeLV, when administered to cats of the target age was determined. Kittens received two vaccinations when aged 7 to 9 weeks old, and were subsequently challenged up to 36 months later with the FeLV-A Glasgow isolate.

**Results:**

In all studies, all of the younger aged control kittens showed persistent FeLV p27 antigenaemia confirming that the challenge virus was severe and efficacious. In contrast, the control cats did not show the required level of persistent antigenaemia, with a maximum of 45% cats affected in the middle duration study and only 10% in the longer study. However, apart from one animal in the short duration study, all of the cats vaccinated with Versifel FeLV were negative for persistent antigenaemia and can be considered treatment successes.

**Conclusion:**

In conclusion, we have shown that although age-related resistance to infection with a virulent FeLV challenge is evident from as early as 10 months of age, vaccination with Versifel FeLV may aid in the protection of cats from FeLV related disease up to three years after primary vaccination as kittens.

## Background

Feline leukaemia virus (FeLV) is a gamma-retrovirus [1] and a significant pathogen of domestic cats throughout the world, and also other small felids such as the wildcat and lynxes [2]. Contact and saliva are the main modes of transmission although faeces have also been shown to contain virus shed from infected cats [3]. Cats exposed to FeLV may become either persistently viraemic or recover from the infection, with young cats being more rapidly infected following contact exposure [4]. A third category of cats infected with FeLV are those with a latent infection; they are not viraemic, but have a covert infection and do not develop FeLV disease [5]. Viraemic animals do not respond to FeLV antigens and show limited response to the virus, and are as a result at higher risk of developing fatal disease. Those cats which do recover from the infection produce virus neutralising antibodies which are considered important in preventing re-infection and can be passed through colostrum to protect neonatal kittens [6]. Antibodies to the feline oncornavirus-associated cell membrane antigen (FOCMA) can also be induced, which protect against the development of FeLV-related neoplastic disease [7]. In addition to antibody-mediated responses the role of cell-mediated immune responses may also be involved with protection.

As FeLV is still a significant pathogen of domestic cats worldwide it is clear that there are demands for methods to protect against infection or virus-related disease. Previous studies reviewed by Sparkes [8] have examined the protection of cats against infection afforded by vaccination with a variety of commercial vaccines, which were shown to vary considerably in efficacy. Another study examined the protection of cats against FeLV challenge following vaccination with Leukocell™ 2 and showed duration of immunity lasting at least 12 months following the primary course [7]. Here we present data from three separate clinical studies examining protection against FeLV-related disease at 8, 20 and 36 months after the primary vaccination regimen.

## Methods

### Experimental design

Three separate studies were conducted; all were established with two treatment groups (control and vaccinates) but as a high level of age related resistance to challenge has been reported [4] a third group of young kittens was added to each study immediately prior to challenge in order to validate the challenge virus titre and associated regimen.

The studies utilised a randomised complete block design, and Tables 1, 2 and 3 illustrate each study design.

**Table 1:** Study design 8 month duration of immunity

**Treatment Group**	**Test Article (TA)**	**Dose Volume TA**	**Days of TA Admin**	**Route of TA**	**Day of FeLV Challenge**	**Volume/ Route of Challenge**	**Number of Animals**
T01	Water for Injection	1 mL	0, 21	SC	243	2 mL/IP	11
T02	Versifel FeLV	1 mL	0, 21	SC	243	2 mL/IP	16
T03	-	-	-	-	243	1 mL/IP	5

**Table 2:** Study design 20 month duration of immunity

**Treatment Group**	**Test Article (TA)**	**Dose Volume TA**	**Days of TA Admin**	**Route of TA**	**Day of FeLV Challenge**	**Volume/ Route of Challenge**	**Number of Animals**
T04	Water for Injection	1 mL	0, 21	SC	631	1 mL/IP	11
T05	Versifel FeLV	1 mL	0, 21	SC	631	1 mL/IP	16
T06	-	-	-	-	631	1 mL/IP	6

**Table 3:** Study design 36 month duration of immunity

**Treatment Group**	**Test Article (TA)**	**Dose Volume TA**	**Days of TA Admin**	**Route of TA**	**Day of FeLV Challenge**	**Volume/ Route of Challenge**	**Number of Animals**
T07	Water for Injection	1 mL	0, 21	SC	1143	1 mL/IP	11
T08	Versifel FeLV	1 mL	0, 21	SC	1143	1 mL/IP	16
T09	-	-	-	-	1143	1 mL/IP	6

Animals were examined for the presence of FeLV p27 antigen in sera collected weekly from three weeks after challenge to 15 week after challenge. The studies were run at a Contract Research Organisation at the University of Lyon, France, and complied with both the institutional operating procedures, appropriate animal welfare legislation and separate Pfizer ethical review processes.

### Animals

All animals were specified pathogen free, European mixed breed cats, 51 to 63 days of age on day 0 (groups T01, T02, T04, T05, T07 and T08) or 72 to 109 days old (groups T03, T06 and T09) prior to challenge, and obtained from a commercial breeder. Cats were equally split between males and females in all treatment groups. Cats were routinely tested by ELISA for the presence or absence of antibodies to the following pathogens – calicivirus, FIV, FeLV, infectious peritonitis, viral rhinotracheitis, panleukopenia, intestinal coronavirus and rotavirus – by a commercial diagnostic laboratory using proprietary assays and analytical methods.

All study animals were negative for FeLV p27 antigen, and anti-FOCMA and gp70 antibodies prior to vaccination and also prior to challenge.

### Vaccine

The vaccine used was an experimental serial of Versifel FeLV, containing inactivated feline leukaemia virus at a minimum dose and a proprietary adjuvant system.

### Challenge material

Prior to administration of the viral challenge, a sufficient volume of the frozen FeLV-A Glasgow [9] isolate stock (mean titre 5.78 x 10^6^ ffu/mL) was thawed in a sterile container. The FeLV challenge suspension for administration to cats was prepared by a 1:10 dilution of the stock virus to a target concentration of 5.78 x 10^5^ ffu/mL using sterile DMEM and HEPES buffers as diluents.

The site for the intraperitoneal injection was the caudolateral aspect of the right ventral abdomen. Using a separately prepared sterile syringe and needle for each cat, and taking care to avoid puncturing internal organs or the intestines, 1 mL of the FeLV challenge suspension was injected into the caudolateral peritoneal cavity of each animal in groups T03 to T09. Cats in groups T01 and T02 received a 2 mL dose. Cats were challenged either 8 months, 20 months or 36 months after vaccination. Previous laboratory studies performed by the authors indicated that this challenge titre, volume and route of administration were sufficient to result in FeLV infection in younger animals.

### Animal observations

All study animals were observed at least once daily for signs of distress or ill health. In addition, cats and kittens had a physical examination prior to enrolment and vaccination (if applicable). Clinical observations, rectal temperature and injection site measurements were also performed following vaccinations and also at the time of sample collection following challenge.

### Sample collection

Blood samples (approximately 2 mL on each occasion) were collected from the jugular vein into plain tubes (without anti-coagulant) using disposable sterile needles. If deemed appropriate by the investigator cats could be sedated prior to sample collection.

Baseline blood samples were collected from each kitten in T01 and T02 on study day -3 and T03 on day 238 prior to challenge. Three weeks after challenge, commencing on day 264, blood samples were collected from all three groups on a weekly basis (271, 278, 285, 292, 299, 306, 313, 320, 327, 334, 341 and 348) until study completion.

Blood samples were collected from each kitten in T04 and T05 on study days −1 and 617 and from group T06 on day 629. Three weeks after challenge, commencing on day 652, blood samples were collected from all three groups on a weekly basis (659, 666, 673, 680, 687, 694, 701, 708, 715, 722, 729 and 736) until study completion.

Blood samples were collected from each kitten in T07 and T08 on study days -1, 100, 982 and 1052 and from kittens in T09 on study day 1136. Three weeks after FeLV challenge, commencing on study day 1164, blood samples were collected from all three groups on a weekly basis (on study days 1164, 1171, 1178, 1185, 1192, 1199, 1206, 1213, 1220, 1227, 1234, 1241 and 1248) until study completion.

Blood samples collected from each animal were stored overnight in a refrigerator and then centrifuged the following day at approximately 3000 rpm for approximately 15 minutes, as per site standard operating procedures. Following centrifugation, serum was harvested from each tube and divided into two aliquots, labelled and stored frozen in cryogenic tubes at ≤ -70 °C. Blood and serum samples were collected and individually labelled. One of the two aliquots of serum was despatched to a Contract Research Laboratory and analysed for FeLV p27-antigen (PetChek FeLV Antigen Test Kit, IDEXX) as per the test kit instructions, with further confirmatory analysis performed on highly positive samples. This assay uses commercial proprietary technology but has a published relative sensitivity of 98.6% and relative specificity of 98.2%. The presence of gp70 antibodies was evaluated by ELISA according to the test kit instructions (FeLV gp70 Antibody ELISA, European Veterinary Laboratory) and anti-FOCMA antibodies were evaluated by IFA according to the analytical laboratory standard operating procedures. For the gp70 antibody ELISA a titre greater than 90 was considered positive while for anti-FOCMA antibodies a titre greater than 20 was considered positive. All assays were validated by the analytical laboratory prior to use in these studies according to their standard operating procedures and in conjunction with Pfizer Animal Health.

### Statistical analysis

#### Primary efficacy variable

The primary efficacy variable was the p27 antigen analysis of serum samples. According to the Monograph 01/2005:1321 Feline Leukaemia Vaccine (Inactivated) only summaries of the data need be presented in terms of the number and percentage of animals with persistent antigenaemia (as determined by p27 protein), by treatment group. Persistent antigenaemia for an animal was defined (as per Pharmacopoeia) as being when an animal was positive for the p27 antigen for three consecutive weeks or on five separate occasions (consecutively or not) from the third week to the fifteenth week after FeLV challenge.

#### Secondary variables

Gp70 antibody and anti-FOCMA antibody results were summarised by treatment group and time by calculating the geometric means, minimums and maximums and categorical results by test result and treatment group.

Body Weight for each day was summarised in terms of mean, standard deviation, minimum and maximum for each treatment group.

Rectal temperature was summarised by calculating descriptive statistics including the mean, standard deviation, minimum and maximum, by treatment group and day.

## Results

### Pre-treatment and pre-challenge FeLV serology status

The geometric mean titres of gp70 antibodies are listed in Table 4 and 5.

**Table 4:** Geometric mean gp70 antibody titre, 20 month DOI

**Day**	**Group**	**<30**	**<30 / 30**	**30**	**30 / <30**	**90 / <30 **	**30 / 90**	**90**	**90 / 30**	**90 / 270**	**270**	**270 / 90**	**270 / ≥810**	**≥810**	**Geometric mean**
−1	T04 (n = 11)	11	0	0	0	0	0	0	0	0	0	0	0	0	10
	T05 (n = 16)	16	0	0	0	0	0	0	0	0	0	0	0	0	10
42	T04 (n = 11)	11	0	0	0	0	0	0	0	0	0	0	0	0	10
	T05 (n = 16)	6	0	2	1	0	0	4	0	0	1	0	1	1	42.3
617	T04 (n = 11)	9	0	1	0	0	0	0	0	0	1	0	1	0	14.9
	T05 (n = 16)	5	1	1	0	1	1	1	2	1	0	1	1	0	36.5
629	T06 (n = 6)	6	0	0	0	0	0	0	0	0	0	0	0	0	10

**Table 5:** Geometric mean gp70 antibody titre, 36 month DOI

**Day**	**Group**	**<30**	**<30 / 30**	**30**	**30 / <30**	**90 / <30 **	**<30 / 90**	**30 / 90**	**90**	**90 / 30**	**90 / 270**	**270**	**270 / 90**	**270 / ≥810**	**≥810**	**Geometric mean**
−1	T07 (n = 11)	11	0	0	0	0	0	0	0	0	0	0	0	0	0	10
	T08 (n = 16)	16	0	0	0	0	0	0	0	0	0	0	0	0	0	10
42	T07 (n = 11)	11	0	0	0	0	0	0	0	0	0	0	0	0	0	10
	T08 (n = 16)	6	0	6	1	0	0	0	0	0	0	1	0	0	2	33.3
100	T07 (n = 11)	10	0	0	0	0	1	0	0	0	0	0	0	0	0	10
	T08 (n = 15)	11	0	0	0	0	0	2	0	0	1	0	0	0	1	20.1
982	T07 (n = 10)	5	2	1	1	0	0	0	1	0	0	0	0	0	0	16.4
	T08 (n = 14)	11	0	0	0	0	0	0	0	0	0	1	0	1	1	22.8
1052	T07 (n = 10)	3	0	2	3	0	0	1	1	0	0	0	0	0	0	21.6
	T08 (n = 14)	10	0	0	1	0	0	0	1	0	0	2	0	0	0	19.5
1136	T07 (n = 10)	4	0	1	0	0	0	1	2	0	0	2	0	0	0	39.5
	T08 (n = 14)	6	0	2	2	0	0	0	2	0	1	1	0	0	0	26.7
	T09 (n = 6)	6	0	0	0	0	0	0	0	0	0	0	0	0	0	10

Baseline serum samples of cats in groups T01, T02, T04, T05, T07 and T08 were negative for FeLV p27 antigen, gp70 antibodies (i.e. ≤90; geometric mean = 10) and anti-FOCMA antibodies (i.e. ≤20; geometric mean = 5; data not shown). Baseline serum samples of the kittens in the additional T03, T06 and T09 groups were negative for FeLV p27 antigen, gp70 antibodies (i.e. ≤90; geometric mean = 10) and anti-FOCMA antibodies (i.e. ≤20; geometric mean = 5; data not shown). Therefore all animals fulfilled the criteria for inclusion in the study as defined in the European Pharmacopeia 5.0 Monograph 01/2005:1321 regarding Feline Leukaemia Vaccine (inactivated).

### Efficacy of protection; p27 antigenaemia

The number and percentage of animals positive for the FeLV p27 antigen at each time point together with the total number and percentage of animals in each treatment group with persistent antigenaemia are summarised in Table 6 (8 month study, groups T01 – T03), Table 7 (20 month study; groups T04 – T06) and Table 8 (36 month study; groups T07 – T09).

**Table 6:** Number and percentage if animals positive for FeLV p27 antigen, and with persistent antigenaemia, 8 month DOI

**Study Day**	**T01 (water)**		**T02 (Versifel FeLV)**		**T03 (no treatment)**	
	**Number of animals**	**Number (%)**	**Number of animals**	**Number (%)**	**Number of animals**	**Number (%)**
−3	11	0 (0.0)	15	0 (0.0)	n/a	n/a
238	11	0 (0.0)	15	0 (0.0)	n/a	n/a
243	n/a	n/a	n/a	n/a	5	0 (0.0)
264	11	5 (45.5)	15	1 (6.7)	5	4 (80.0)
271	11	5 (45.5)	15	1 (6.7)	5	4 (80.0)
278	11	4 (36.4)	15	1 (6.7)	5	4 (80.0)
285	11	5 (45.5)	15	1 (6.7)	5	4 (80.0)
292	11	4 (36.4)	15	1 (6.7)	5	4 (80.0)
299	11	4 (36.4)	15	1 (6.7)	5	4 (80.0)
306	11	4 (36.4)	15	1 (6.7)	5	4 (80.0)
313	11	4 (36.4)	15	1 (6.7)	5	4 (80.0)
320	11	4 (36.4)	15	1 (6.7)	5	4 (80.0)
327	11	4 (36.4)	15	1 (6.7)	5	4 (80.0)
334	11	4 (36.4)	15	1 (6.7)	5	4 (80.0)
341	11	4 (36.4)	15	1 (6.7)	5	4 (80.0)
348	11	4 (36.4)	15	1 (6.7)	5	4 (80.0)
**Number and percentage of animals with persistent antigenaemia**						
	11	4 (36.4)	15	1 (6.7)	5	4 (80.0)

**Table 7:** Number and percentage if animals positive for FeLV p27 antigen, and with persistent antigenaemia, 20 month DOI

**Study Day**	**T04 (water)**		**T05 (Versifel FeLV)**		**T06 (no treatment)**	
	**Number of animals**	**Number (%)**	**Number of animals**	**Number (%)**	**Number of animals**	**Number (%)**
−1	11	0 (0.0)	16	0 (0.0)	n/a	n/a
617	11	0 (0.0)	15	0 (0.0)	n/a	n/a
629	n/a	n/a	n/a	n/a	6	0 (0.0)
652	11	6 (54.5)	15	2 (13.3)	6	6 (100.0)
659	11	6 (54.5)	15	2 (13.3)	6	6 (100.0)
666	11	5 (45.5)	15	0 (0.0)	6	6 (100.0)
673	11	3 (27.3)	15	0 (0.0)	6	6 (100.0)
680	11	3 (27.3)	15	0 (0.0)	6	6 (100.0)
687	11	3 (27.3)	15	0 (0.0)	6	6 (100.0)
694	11	3 (27.3)	15	0 (0.0)	6	6 (100.0)
701	11	3 (27.3)	15	0 (0.0)	6	6 (100.0)
708	11	3 (27.3)	15	0 (0.0)	6	6 (100.0)
715	11	3 (27.3)	15	0 (0.0)	6	6 (100.0)
722	11	3 (27.3)	15	0 (0.0)	6	6 (100.0)
729	11	3 (27.3)	15	0 (0.0)	6	6 (100.0)
736	11	3 (27.3)	15	0 (0.0)	6	6 (100.0)
**Number and percentage of animals with persistent antigenaemia**						
	11	5 (45.5)	15	0 (0.0)	6	6 (100.0)

**Table 8:** Number and percentage if animals positive for FeLV pp27 antigen, and with persistent antigenaemia, 36 month DOI

**Study Day**	**T07 (water)**		**T08 (Versifel FeLV)**		**T09 (no treatment)**	
	**Number of animals**	**Number (%)**	**Number of animals**	**Number (%)**	**Number of animals**	**Number (%)**
−1	11	0 (0.0)	16	0 (0.0)	n/a	n/a
100	11	0 (0.0)	15	0 (0.0)	n/a	n/a
982	10	0 (0.0)	14	0 (0.0)	n/a	n/a
1052	10	0 (0.0)	14	0 (0.0)	n/a	n/a
1136	10	0 (0.0)	14	0 (0.0)	6	6 (100.0)
1164	10	1 (10.0)	14	0 (0.0)	6	6 (100.0)
1171	10	1 (10.0)	14	0 (0.0)	6	6 (100.0)
1178	10	1 (10.0)	14	0 (0.0)	6	6 (100.0)
1185	10	1 (10.0)	14	0 (0.0)	6	6 (100.0)
1192	10	0 (0.0)	14	0 (0.0)	6	6 (100.0)
1199	10	1 (10.0)	14	0 (0.0)	6	6 (100.0)
1206	10	1 (10.0)	14	0 (0.0)	6	6 (100.0)
1213	10	1 (10.0)	14	0 (0.0)	6	6 (100.0)
1220	10	1 (10.0)	14	0 (0.0)	6	6 (100.0)
1227	10	1 (10.0)	14	0 (0.0)	6	6 (100.0)
1234	10	1 (10.0)	14	0 (0.0)	6	6 (100.0)
1241	10	1 (10.0)	14	0 (0.0)	6	6 (100.0)
1248	10	1 (10.0)	14	0 (0.0)	6	4 (66.7)
**Number and percentage of animals with persistent antigenaemia**						
	10	1 (10.0)	14	0 (0.0)	6	6 (100.0)

#### Eight month challenge

In treatment group T01 (water for injection), six individual cats were seen to be positive for FeLV p27 antigen on one or more occasions after administration of the FeLV viral challenge on study day 243; of these six animals, four met the criteria for persistent antigenaemia. The remaining five cats in T01 were negative for FeLV p27 antigen throughout the period of assessment. Thus the rate of persistent infection with FeLV in T01 was 36.4% (four out of 11 cats).

Of the 15 cats enrolled in group T02 (Versifel FeLV) only one animal was observed to be positive for FeLV p27 antigen post-challenge; this cat was positive on each sampling occasion from study day 264 to 348 (inclusive) and therefore met the criteria for persistent antigenaemia. Thus for group T02, where 14 out of 15 cats treated with Versifel FeLV did not develop any signs of infection with the FeLV virus, the treatment success rate was 93.3%.

Four out of five (80.0%) of the kittens enrolled in T03 and which had received no treatment, were observed to be positive for FeLV p27 antigen on each post challenge sampling occasion and therefore these four animals each met the criteria for persistent antigenaemia.

#### 20 Month challenge

In group T04 (water for injection), six of the 11 cats were positive for FeLV p27 antigen on day 652, three weeks after challenge. Of these six, three cats remained positive for FeLV p27 antigen until the end of the study. Of the 11 animals in this treatment group five (45.5%) met the criteria for persistent antigenaemia.

Of the 16 cats enrolled in group T05 (Versifel FeLV) one died before challenge and analysis of the serum from the remaining 15 cats indicated that two were positive for FeLV p27 antigen three weeks after challenge, but thereafter remained negative until study completion. All other cats were negative throughout the study. Overall, none of the cats met the criteria for persistent antigenaemia and consequently all were considered treatment successes.

All of the six kittens enrolled in group T06, which received no treatment were positive for FeLV p27 antigen on each occasion on which serum samples were collected. Consequently, all six cats in this treatment group met the criteria for persistent p27 antigenaemia.

#### 36 Month challenge

In treatment group T07 (water for injection) one cat was euthanased on study day 213suffering from dyskinesia and of the ten cats remaining one was positive for FeLV p27 antigen on study day 1164, three weeks after challenge, and remained positive for the remainder of the study. Of the ten cats in this treatment group only one (10%) met the criteria for persistent antigenaemia.

In treatment group T08 (Versifel FeLV) one cat died on study days 457 and another was euthanased on study day 91; neither of these was related to FeLV disease. Of the remaining 14 cats none were positive for FeLV p27 antigen on study day 1164, three weeks after challenge, and all remained negative until the end of the study. Overall none of the cats in this treatment group met the criteria for persistent p27 antigenaemia and consequently all 14 animals (100%) were considered to be treatment successes.

In the kitten control group T09 (no treatment), all six kittens were positive for FeLV p27 antigen on study day 1164, three weeks after challenge, and four remained positive on all sampling points until the end of the study. Two kittens were negative on study day 1248 only. Consequently, all six cats in this treatment group (100%) met the criteria for persistent antigenaemia thus validating the challenge phase of the study.

#### Gp70 Antibody and anti-FOCMA serology

Summaries of the gp70 antibody titres and the geometric means are shown in Tables 4 and 5.

#### 20 Month challenge

Ten of 11 cats in group T04 had gp70 antibody titres of ≤30 on day −1, 42 and 617. One cat had a gp70 titre of 270 on day 617, prior to challenge of day 633 although this is considered to be anomalous as it was negative for FeLV antigen at the same sampling time point.

All of the cats in group T05 were seronegative on day −1, but by day 42 three of 16 (19%) had antibody titres >90 and were considered seropositive, with a group geometric mean of 42.3. Two of these cats had antibody titres that returned to <90 by day 617, while the third had a titre if 270/90. Two cats which were negative on day −1 and 42, had titres >90 on day 617, possibly indicating a slowly developing antibody response.

All six cats in group T06 were seronegative on day 629, with gp70 antibody titres <30.

#### 36 Month challenge

All 11 cats in group T07 had gp70 antibody titres ≤30 on study days −1, 42 and 100, although one cat at the latter time point had a titre of <30/90. Nine cats, of the 10 remaining, had gp70 antibody titres ≤30 on study day 982 with one cat having a titre of 90 and eight cats having titres ≤30 on study day 1052 with one cat having a titre of 90 and another a titre of <30/90. Five cats had a gp70 antibody titre ≤30 on study day 1136 with one cat having a titre of 30/90, two cats having a titre of 90 and two cats having a titre of 270.

All of the 16 cats in group T08 were seronegative on study day −1 (gp70 antibody titres ≤30). By study day 42 (three weeks post completion of the primary vaccination course) three of the 16 cats (19%) had gp70 antibody titres >90 and were therefore considered to be seropositive, with a group geometric mean of 33.3. By study day 100 two of the remaining 15 cats (13%) had gp70 antibody titres >90, with a group geometric mean of 20.1. On study day 982 three of the remaining 14 cats (21%) had a gp70 antibody titre >90, with a group geometric mean of 22.8. On study day 1052 two of the 14 cats (14%) had gp70 antibody titres >90, with a group geometric mean of 19.5. On study day 1136 two of the 14 cats (14%) had gp70 antibody titres >90, with a group geometric mean of 26.7.

All six cats in group T09 were seronegative on study day 1136, with gp70 antibody titres ≤30.

### Bodyweights

Although there was some variation in the body weights of cats, descriptive data summaries showed little apparent difference within each individual study between adult control and vaccinated cats over the measured period around vaccination and the period associated with FeLV challenge.

### Rectal temperatures

Descriptive data summaries showed little apparent difference within each individual study between adult control and vaccinated cats in rectal temperature over the measured period around vaccination and the period associated with FeLV challenge.

## Discussion

These studies, which were conducted according to the requirements defined in European Pharmacopeia 5.0, Monograph 01/2005:1321 regarding Feline Leukaemia Vaccine (Inactivated), evaluated protection against a FeLV challenge provided by Versifel FeLV over an extended period. Animals were slightly younger than the target age when first vaccinated with Versifel FeLV (between seven and eight weeks of age) and were challenged with virulent FeLV either eight months, 20 months or 36 months after the vaccination course. Thus, at the time of FeLV challenge, cats in the adult groups were aged approximately 10 months, 22 months or 38 months old, while the kitten groups were approximately three to four months old. The monograph specifies that for a successful challenge study, the challenge virus must be an epidemiologically relevant isolate, and that less than 20% of vaccinated cats should develop persistent infection and more than 80% of control cats must develop persistent infection.

When referring to the monograph requirements none of the three studies was a complete success, even though they all used the same vaccine. The vaccinated groups did not show persistent antigenaemia and thus it could be considered that the vaccine offered full protection against a subsequent challenge with virulent FeLV during the timescale of each study. This observation is offset by the fact that the adult control animals in each study did not succumb to the challenge infection in sufficient numbers with a maximum of 45.5% animals in the 20 month duration study showing persistent antigenaemia. Given that the FeLV challenge was administered to these cats when they were approximately 10 to 39 months of age, it is likely that some degree of age related immunity to FeLV infection may have been a factor in this low rate of persistent p27 antigenaemia in unvaccinated cats. Age related resistance to FeLV challenge has been reported previously [4,6] and consequently this result was not unexpected. Indeed, one study by Hoover and others [10] showed a rapid decrease in susceptibility to experimental infection with FeLV, with 100% of newborn kittens able to be infected compared to 15% of cats aged 4 months or 1 year old. A recent study comparing FeLV vaccine efficacy challenged cats with a considerably smaller dose volume and titre and achieved persistent antigenaemia in seven of eight controls, as well as five of eight vaccinated cats at seven months of age [11]. The high rate of p27 antigenaemia observed in the additional kitten control groups, which were approximately 12 to 16 weeks of age when subjected to the same FeLV challenge as the older cats and at the same time, provides evidence that the chosen dose and route of administration for the FeLV material resulted in an effective and aggressive challenge.

The anomalous gp70 antibody result seen in two cats in treatment group T07 on study day 1136 may raise the question of whether the cats might have been exposed to extraneous FeLV during the post vaccination period. This is considered to be extremely unlikely as these observations were only made in a minority of the cats and similar results were observed in group T08 (two vaccinates which had until day 1136 either remained seronegative or had been seropositive on just one occasion post vaccination). Furthermore, all cats in groups T07 and T08 remained healthy throughout the pre-challenge period and were confirmed to be negative for p27 antigen when tested at various intervals prior to challenge. The cats were obtained from a Specified Pathogen Free (SPF) facility and were housed in a bio-security category 2 facility throughout the duration of the study; it would therefore be extremely unlikely that extraneous FeLV virus could have gained access into the facility.

Products of the viral envelope gene, and specifically the major envelope protein gp70, are thought to be involved in virus host range recognition functions, and as such are also major targets for antibody-mediated virus neutralisation [12]. However, antibodies against gp70 are generally not induced following vaccination [7], an observation which appears to correlate with findings seen in this study. This suggests that beside antibody seroconversion additional immunological factors (such as cell-mediated immunity) should be considered in the vaccine-induced duration of immunity against FeLV. Indeed, other studies have shown a role for cell-mediated immunity in protection against FeLV. Flynn and others [13] showed that following vaccination of cats with a FeLV DNA vaccine containing the *gag*/*pol* and *env* genes from the FeLV Glasgow A strain there was a higher level of virus-specific effector cytotoxic T lymphocytes in the peripheral blood and lymphoid organs than observed in unvaccinated control, persistently viraemic cats. The apparent absence of viraemia in cats following vaccination with the Versifel FeLV vaccine, in that FeLV antigen could not be detected in serum samples following challenge, does not mean that infection was prevented as clinical observations showed enlarged lymph nodes indicative of FeLV-related disease. Other studies have shown that during an asymptomatic phase of infection that no severe clinical signs were observed, with some cats showing mild stomatitis or gingivitis, diarrhoea and mild weight loss [14], which is similar to what was observed in the current study. The correlation of protection with low level virus neutralising gp70 antibodies only seen in a small number of vaccinated cats is an observation which has been reported previously. A recent study [11] examined the immunity to FeLV induced by vaccination with a variety of inactivated viruses. Two of the vaccines were found to protect against subsequent challenge, but this effective containment of the virus occurred despite minimal detectable virus neutralising antibodies being seen.

## Conclusion

In conclusion, we have shown that although age-related resistance to infection with a virulent FeLV challenge is evident from as early as 10 months of age, vaccination with Versifel FeLV can help protect cats from FeLV related disease up to three years after primary vaccination as kittens.

## Competing interests

The authors declare they have no competing interests. All are employed by Pfizer Animal Health.

## Authors’ contributions

SW was responsible for writing the manuscript, data interpretation and conclusions. AS, JT and TC were responsible for vaccine development; LB, CH and GN were responsible for assay development and technical development; JG provided regulatory support; and JT and GS also provided project management support. All authors read and approved the final manuscript.
